# Collagen Coatings on Titanium: Comparative Study of Electrochemical and Chemical Formation

**DOI:** 10.3390/ma19214748

**Published:** 2026-11-08

**Authors:** Marin Hodalj, Ines Despotović, Antonio Zandona, Maja Katalinić, Maja Ivanić, Jozefina Katić, Željka Petrović

**Affiliations:** 1 Department of Electrochemistry, Faculty of Chemical Engineering and Technology, University of Zagreb, 10000 Zagreb, Croatia; hodaljmarin@gmail.com (M.H.); jkatic@fkit.unizg.hr (J.K.); 2 Division of Physical Chemistry, Ruđer Bošković Institute, 10002 Zagreb, Croatia; 3 Division of Toxicology, Institute for Medical Research and Occupational Health, 10001 Zagreb, Croatia; azandona@imi.hr (A.Z.); mkatalinic@imi.hr (M.K.); 4 Division for Marine and Environmental Research, Ruđer Bošković Institute, 10002 Zagreb, Croatia; maja.ivanic@irb.hr; 5 Division of Materials Chemistry, Ruđer Bošković Institute, 10002 Zagreb, Croatia

**Keywords:** titanium, collagen, cyclic voltammetry, self-assembly, DFT, cytocompatibility

## Abstract

This study investigates the influence of two collagen coating formation approaches, self-assembly and electrochemical formation by cyclic voltammetry, on the surface properties and electrochemical behavior of titanium (Ti). Collagen-modified Ti surfaces were characterized before and after 7 days of immersion in Fusayama artificial saliva using attenuated total reflectance Fourier-transform infrared spectroscopy, field emission scanning electron microscopy coupled with energy-dispersive X-ray spectroscopy, X-ray diffraction, and electrochemical impedance spectroscopy. Self-assembly produced a continuous collagen layer, whereas cyclic voltammetry generated a fibrillar coating. During immersion, calcium phosphate (CaP) deposits formed on both collagen-modified surfaces, but with distinctly different spatial organization. The self-assembled coating underwent reorganization, whereas the electrochemically formed coating retained its fibrillar framework closely associated with the CaP deposits. This organic–inorganic architecture was accompanied by enhanced electrochemical barrier properties after 7 days compared with the self-assembled coating. Density functional theory calculations further showed that one-electron oxidation of an adsorbed collagen-model tripeptide induces pronounced structural rearrangement, providing molecular-level insight into the redox response of collagen-derived species at the TiO_2_ interface. In addition, prolonged exposure of Ti surfaces to cell culture media resulted in negligible Ti release, while the resulting conditioned media did not reduce the metabolic viability of HGF-1 or MG-63 cells under the investigated conditions, supporting favorable indirect in vitro cytocompatibility.

## 1. Introduction

The surface properties of implant materials play a critical role in the initial interactions with the surrounding biological environment. For titanium (Ti), widely used in dental implantology, physicochemical surface characteristics strongly influence cell–material interactions and subsequent tissue responses. Early studies demonstrated that Ti surface roughness can alter the response of osteoblast-like cells [1] and influence bone–implant contact in vivo [2]. These findings have motivated the development of various surface modification and functionalization strategies aimed at tailoring the implant–tissue interface and enhancing its biological performance [3].

Surface modification techniques are broadly categorized into physical, chemical, and biological approaches, each providing different means of tailoring the implant surface and its interaction with the surrounding tissue [3,4]. Physical and chemical treatments, such as sandblasting, acid etching, and micro-arc oxidation, are used to modify the surface topography, morphology, and physicochemical properties of Ti [5,6]. In addition to these treatments, functional coatings provide an alternative approach to tailor the implant–tissue interface by introducing bioactive components that can influence cellular responses [4]. Such coatings include inorganic materials, particularly calcium phosphates [7], as well as bioactive organic components, including growth factors such as bone morphogenetic proteins [8] and extracellular matrix (ECM) proteins such as collagen [9].

Among biomolecules used for surface functionalization, collagen, a major structural component of the extracellular matrix, is of particular interest for the biofunctionalization of Ti surfaces. Collagen-based coatings have shown potential to promote osseointegration [10]. In vivo studies have shown that type I collagen-functionalized Ti surfaces can enhance early bone formation and osteogenic differentiation [11], improve osseointegration [12], and promote bone maturation and mineralization at the implant–bone interface [13].

The method used to form collagen coatings on Ti surfaces can influence the coating’s stability and interfacial properties [14]. Different strategies have been developed, including physical adsorption, self-assembly, covalent immobilization, and electrically assisted approaches [14,15,16,17]. Physical adsorption represents a relatively simple approach, whereas covalent immobilization generally requires prior surface activation and chemical functionalization. Marín-Pareja et al. compared physical adsorption of type I collagen on activated Ti surfaces with covalent immobilization through organosilane chemistry and showed that the amount and stability of immobilized collagen depended on both the immobilization method and the surface activation treatment [14]. Gregurec et al. prepared collagen–alginate films on titania by layer-by-layer self-assembly followed by chemical crosslinking, resulting in stable coatings with a fibrillar morphology [15]. In our previous study, hydrolyzed collagen alone was self-assembled on Ti from an aqueous solution followed by post-thermal treatment, and the resulting coating exhibited high electrochemical barrier properties in artificial saliva [16].

Electrically assisted deposition has also been explored for collagen coating formation on Ti. Wang et al. employed electrophoretic deposition (EPD) to form a type I collagen coating on Ti, which was subsequently subjected to biomimetic mineralization [17]. Potential-controlled electrodeposition has also been employed for collagen immobilization on Ti. Kamata et al. investigated collagen electrodeposition under different pH, potential, and deposition-time conditions and found that alternating-potential deposition between −1 and +1 V vs. the saturated calomel electrode (SCE) produced a durable and relatively uniform collagen layer compared with conventional immersion [18]. More recently, electrochemical deposition of collagen fibrils on Ti was shown to provide high electrochemical barrier properties and to promote calcium phosphate nucleation in artificial saliva [19].

Although electrochemical methods have been used for collagen coating formation, cyclic voltammetry, to the best of our knowledge, has not previously been explored for this purpose on Ti surfaces. At the same time, molecular-level understanding of electrochemically assisted collagen coating formation remains limited, despite recent efforts to correlate experimental observations with theoretical calculations [19]. Moreover, although immersion-based and electrochemically assisted approaches to collagen immobilization have been reported, a direct comparison of collagen coatings formed by self-assembly and cyclic voltammetry, including their structural differences, mineralization and electrochemical behavior in artificial saliva, is still lacking.

Therefore, this study compares collagen coatings formed on Ti by self-assembly and cyclic voltammetry, examining how the formation route influences coating architecture and how the resulting coatings subsequently evolve during exposure to artificial saliva. Surface, structural, and electrochemical characterization was complemented by density functional theory (DFT) calculations examining the redox behavior and oxidation-induced structural changes in an adsorbed collagen-model tripeptide at the TiO_2_ interface. Ti release during prolonged exposure to cell culture media and indirect cytocompatibility were additionally evaluated on human gingival fibroblasts and osteoblast-like cells to provide an initial assessment of the biological response under the investigated conditions.

## 2. Materials and Methods

### 2.1. Materials, Chemicals and Solutions

Titanium (Ti) rods, cut into disks (⌀ = 12 mm; 99.9%; Alfa Aesar^®^, Karlsruhe, Germany), served as substrates for the collagen coating. Before coating formation, the Ti surface was mechanically abraded with SiC emery papers of grit sizes #180, #240, #500, and #600, ultrasonically cleaned for 5 min with absolute ethanol (p.a.; Gram-Mol^®^, Zagreb, Croatia), acetone (p.a.; Gram-Mol^®^, Zagreb, Croatia), and MilliQ water (Hydrolab, Wesoła, Straszyn, Poland, 0.05 μS/cm). The Ti substrates were then dried in a nitrogen stream (99.999%, Messer, Germany) and exposed to air for 10 min before coating formation.

An aqueous solution of bovine-derived hydrolyzed collagen (HC; approximately 45 mg mL^−1^, corresponding to a nominal concentration of 10^−2^ mol dm^−3^ based on an estimated average molar mass of 4.5 kDa; Medex d.o.o., Ljubljana, Slovenia) was used for coating preparation. The pH of the solution was 4.4.

Fusayama artificial saliva was used as a model oral electrolyte throughout the immersion experiments and it was prepared by dissolving 0.40 g NaCl, 0.40 g KCl, 0.60 g CaCl_2_·2H_2_O, 0.58 g Na_2_HPO_4_·2H_2_O, and 1.00 g urea in MilliQ water and adjusting the final volume to 1 L. The pH was 6.8 [20].

### 2.2. The Preparation of Collagen Coatings on Ti Substrates

The collagen coating was formed in two ways: (i) chemically by self-assembly and (ii) electrochemically by cyclic voltammetry at 25 ± 3 °C.

*Chemical formation by self-assembly*. For the coating prepared by self-assembly, the Ti disks were immersed in the hydrolyzed collagen solution for 24 h. The Ti samples modified by self-assembly are labeled Ti/collagen SAM.

*Electrochemical formation by cyclic voltammetry*. The electrochemical formation of the collagen coating was carried out in a three-electrode cell, with the Ti sample (an exposed area of 1 cm^2^) as the working electrode, Ag/AgCl/3 M KCl as the reference electrode with a potential of 0.210 V versus the standard hydrogen electrode, and a graphite rod as the counter electrode. The potential range for coating formation was selected based on the cyclic voltammogram of Ti recorded in the HC solution. The initial cyclic voltammogram was recorded over the potential range from −0.3 to 0.5 V at 50 mV s^−1^, and the potential range from −0.3 to 0.1 V was selected for coating formation. The collagen coating was formed by cycling the Ti electrode between −0.3 and 0.1 V, starting and ending at the open-circuit potential (OCP), at a scan rate of 10 mV s^−1^ for 20 cycles. The measurements were performed using an Autolab 128 N potentiostat/galvanostat (Metrohm Autolab B.V., Utrecht, The Netherlands) controlled by Nova^®^ 2.1.8 software (Metrohm Autolab B.V., Utrecht, The Netherlands). The samples are labeled Ti/collagen CV. After coating formation, the samples were briefly rinsed by immersion in water and then allowed to dry in air.

### 2.3. Characterization of the Unmodified and Collagen-Modified Ti Substrates

For each experimental group (bare Ti, Ti/collagen SAM, and Ti/collagen CV), three independently prepared samples were examined. Comparable responses were obtained within each group, and representative responses are presented in the manuscript.

The morphological characteristics of unmodified and modified Ti substrates were investigated using field emission scanning electron microscopy (FE-SEM, model JSM-7000F, JEOL Ltd., Tokyo, Japan) in conjunction with an EDS/INCA 350 energy dispersive X-ray analyzer (Oxford Instruments, High Wycombe, UK) to determine the elemental composition of the Ti surfaces. Since the energies of the N Kα (0.392 keV) and Ti Lα (0.452 keV) [21] lines are close to each other, an accelerating voltage of 5 kV was used to reduce the contribution of the Ti substrate and enhance the relative contribution of the near-surface collagen layer.

The chemical characteristics of the unmodified and modified Ti surfaces were monitored using attenuated total reflection Fourier transform infrared spectroscopy (ATR-FTIR). Spectra were recorded with the Frontier spectrometer (PerkinElmer, Waltham, MA, USA) from 4000 to 400 cm^−1^ at a resolution of 4 cm^−1^ and 20 scans per measurement.

The phase composition of the deposits on unmodified and modified Ti surfaces after 7 days of exposure to artificial saliva was analyzed using the Empyrean X-ray diffractometer, model 3 (XRD, Malvern Panalytical B.V., Almelo, The Netherlands), in the 2*θ* range between 10° and 90°, at 45 kV and 40 mA, with CuKα radiation (λ = 0.1542 nm).

The influence of collagen coating on the electrochemical behavior of Ti in Fusayama artificial saliva was investigated by electrochemical impedance spectroscopy (EIS) at the OCP. Before EIS measurements, the samples were allowed to stabilize at OCP for 1 h. The measurements were performed in the same three-electrode cell configuration used for electrochemical coating formation, with the Ti sample (an exposed area of 1 cm^2^) as the working electrode, Ag/AgCl/3 M KCl as the reference electrode, and a graphite rod as the counter electrode. EIS spectra were recorded in the frequency range from 10^5^ to 10^−3^ Hz using an AC amplitude of 10 mV with a PalmSens4 potentiostat/galvanostat/frequency analyzer and PSTrace 5.13 software (PalmSens BV, Houten, The Netherlands). The EIS data were fitted using the same software. The relative fitting errors of the individual equivalent-circuit parameters were below 5%, and the corresponding χ^2^ values are reported in Table 1.

### 2.4. The Quantum-Chemical DFT Calculations

To model a hydrolyzed collagen molecule, a functional tripeptide fragment, Gly–Pro–Hyp was used, following our previously established computational approach [19]. Based on the charge characteristics of hydrolyzed collagen at the experimental solution pH and its previously observed anodic deposition behavior [19], the tripeptide was modeled in its anionic form, NH_2_–Gly–Pro–Hyp–COO^−^. As in our previous computational study [19], a (TiO_2_)_10_ nanocluster, originally employed by Qu and Kroes [22], was used to model the native titanium oxide surface.

The molecular-level insight into the electrochemical oxidation of the collagen-derived tripeptide on the titanium surface, its redox properties and structural changes upon oxidation was obtained by utilizing quantum chemical DFT calculations [23,24]. The relevant TiO_2_/tripeptide configurations were optimized by employing the M06 functional developed by Truhlar’s group [25,26,27] with the 6-31+G(d,p) + LANL2DZ basis set. Pople’s 6-31+G(d,p) double-ξ basis set was chosen for the H, C, O and N atoms, and the LANL2DZ basis set was chosen for the transition metal (Ti) atoms [28]. The configuration of the TiO_2_ cluster was kept fixed during the geometry optimizations of both the anionic and radical species. All the calculated structures were verified to be true minima on the potential energy surface via vibrational frequency analysis under the harmonic oscillator approximation, and the thermal correction to the Gibbs free energy was derived from the same vibrational analysis. The final energy calculations were performed by utilizing a highly flexible 6-311++G(2df,2pd) basis set for the H, C, O and N atoms, whereas the same LANL2DZ ECP-type basis set was used for the titanium atoms. The solvation effects were taken into account by employing the polarizable continuum solvation model SMD (Solvation model based on density) [29]. The water solvent is represented by a dielectric constant of ε = 78.3553. All calculations were carried out using the program package Gaussian 16 (revision C.01) [30].

The topological analysis of the charge density distribution was accomplished by means of Bader’s quantum theory of atoms in molecules (QTAIM) [31] using the program package AIMALL [32] and the SMD/M06/6-31+G(d,p) + LANL2DZ wave function obtained from the optimization.

The reaction Gibbs free energy, ∆_r_G*, was calculated via the equation ∆_r_G* = G*_AB_ − G*_A_ − G*_B_, where G*_AB_ is the total free energy of the resulting AB structure and G*_A_ and G*_B_ are the total free energies of the interacting units A and B, respectively.

The equilibrium redox potential was calculated according to the equation: *E* = −∆_r_G*/nF where ∆_r_G* is the Gibbs free-energy change, n is the number of transferred electrons, and F is the Faraday constant (96.485 C/mol). Following the reduction convention for reporting electrode potentials [33] the (TiO_2_)_10_—tripeptide^●^/(TiO_2_)_10_—tripeptide^−^ redox couple was represented by the reduction half-reaction defined as: (TiO_2_)_10_—tripeptide^●^ + e^−^ → (TiO_2_)_10_—tripeptide^−^. The calculated potential was referenced to the Ag/AgCl/3 M KCl reference electrode using the absolute potential of the standard hydrogen electrode (4.44 V) [34] and the standard potential of the Ag/AgCl/3 M KCl electrode +0.210 V vs. SHE [35].

### 2.5. Chemical Stability and Cytocompatibility Assessment

Based on a literature report in which collagen-coated Ti surfaces were subjected to gamma irradiation at 12 kGy before in vitro experiments [36], the effect of gamma irradiation on the collagen SAM and CV coatings was evaluated at doses of 8, 10, and 15 kGy using a Cobalt-60 gamma source located at the Radiation Chemistry and Dosimetry Laboratory of the Ruđer Bošković Institute. The surface morphology was examined by SEM before and after irradiation, and no evident morphological changes in the coatings were observed at any of the investigated doses. Therefore, the highest tested dose, 15 kGy, was selected for sterilization of the samples used in the subsequent cell-based experiments.

The Ti/collagen CV samples were then incubated in two cell culture media (Dulbecco’s Modified Eagle’s Medium (DMEM) and Eagle’s Minimum Essential Medium (EMEM), Sigma-Aldrich, Steinheim, Germany) either supplemented with fetal bovine serum (FBS, Sigma-Aldrich, Steinheim, Germany) or under serum-free conditions for 90 days. Aliquots of the conditioned media were collected after 1, 4, 7, 14, 21, 60, and 90 days of incubation and subsequently analyzed by inductively coupled plasma mass spectrometry (8900 ICP-QQQ, Agilent, Santa Clara, CA, USA) to assess Ti release into the culture medium and for the assessment of cell viability. For ICP-QQQ analysis, samples were acidified with HNO_3_ (65%, Suprapur, Merck, Darmstadt, Germany) and left overnight before being diluted to a final volume of 10 mL with Milli-Q water. Indium (50 μg L^−1^) was used as an internal standard.

Human gingival fibroblasts (HGF-1, CRL-2014™, ATCC, Manassas, VA, USA) and human osteoblast-like cells (MG-63, CRL-1427™, ATCC, Manassas, VA, USA) were cultured in Dulbecco’s Modified Eagle’s Medium (DMEM) or Eagle’s Minimum Essential Medium (EMEM) supplemented with 10% (*v*/*v*) fetal bovine serum (FBS), and 1% (*v*/*v*) penicillin/streptomycin (PenStrep solution), respectively. The mentioned culture reagents were obtained from Sigma-Aldrich, Steinheim, Germany. Cell cultures were grown at 37 °C in a 5% CO_2_ atmosphere. The growth medium was replaced every two days. The passage of cells was performed in line with the manufacturer’s recommendations. Cells were detached with 0.25% trypsin/EDTA solution (Sigma-Aldrich, Steinheim, Germany), resuspended, and seeded 24 h before experiments. The viability assay (CellTiter-Glo^®^ Luminescent Cell Viability Assay, Promega, Madison, WI, USA) followed the procedure presented previously [37]. Briefly, 10,000 cells per well were seeded in 96-well opaque plates and exposed for 24 h to conditioned media, either serum-free or FBS-supplemented, collected at defined time points of the incubation period from unmodified and collagen-modified titanium (Ti/collagen CV) samples as described in the previous paragraph. After 24 h exposure of cells, the reagent was added, and luminescence was recorded using a microplate reader (SpectraMax^®^ iD3, Molecular Devices, San Jose, CA, USA). Data were obtained from two independent experiments, each performed in duplicate, and expressed as a percentage of viable cells relative to untreated control cells. Statistical analysis was performed using ordinary one-way analysis of variance (ANOVA) followed by Dunnett’s multiple comparisons test. Differences were considered statistically significant at *p* < 0.05.

### 2.6. Use of Generative Artificial Intelligence

During the preparation of this manuscript, the authors used OpenAI (San Francisco, CA, USA) ChatGPT (GPT-5.6 Sol) for the visual composition and design of the Graphical Abstract based on the original concept and scientific content provided by the authors. All experimental data presented in the Graphical Abstract, including SEM micrographs and electrochemical impedance spectroscopy results, were generated by the authors and incorporated without alteration. The authors reviewed and edited the output and take full responsibility for the content of this publication.

## 3. Results and Discussion

### 3.1. Electrochemical Formation of the Collagen Coating (Ti/Collagen CV)

The potential range for collagen CV coating formation was selected based on a cyclic voltammogram of Ti recorded in the HC solution over a wider potential window (−0.3 to +0.5 V) at a scan rate of 50 mV s^−1^ (Figure 1a). Within this voltammogram, the potential range from −0.3 to +0.1 V was characterized by relatively low current densities and the absence of the pronounced anodic current increase observed at more positive potentials. This range was therefore selected for subsequent collagen coating formation by cyclic voltammetry, using 20 cycles at a scan rate of 10 mV s^−1^ (Figure 1b).

### 3.2. Molecular-Level Insight into the Redox Behavior and Structural Response of the Adsorbed Collagen Model

To gain molecular-level insight into the redox behavior and potential oxidation-induced structural changes in hydrolyzed collagen-derived species, DFT calculations were performed. The oxidation of the modeled anionic tripeptide (NH_2_-Gly-Pro-Hyp-COO^−^) adsorbed on a TiO_2_ cluster may represent not only an electron-transfer process but also a chemical activation step facilitating intermolecular coupling. Upon one-electron oxidation, the anionic tripeptide formally gives rise to a neutral radical species that exhibits pronounced changes in molecular geometry. The calculated oxidation potential of the model system was +0.458 V vs. Ag/AgCl/3 M KCl, which is more positive than the upper vertex potential (+0.1 V) applied during collagen coating formation (Figure 1b). Accordingly, the calculated oxidation potential cannot be directly associated with coating formation under the experimental conditions. Nevertheless, the calculations predict pronounced geometric changes upon one-electron oxidation of the model tripeptide. These results provide theoretical insight into the potential structural changes induced by oxidation of a collagen-derived fragment.

A particularly relevant structural change is the substantial elongation of the C–C bond linking the hydroxyproline ring to its carboxylate carbon, from 1.534 Å in the anionic state to 2.988 Å in the radical state (Figure 2). This elongation, accompanied by a rearrangement of the COO^−^ group, i.e., its substantial linearization, may increase the accessibility of the carboxylate moiety and facilitate its interaction with the nucleophilic nitrogen atom of the terminal amino group of a neighboring tripeptide. Importantly, AIM analysis indicates that the C–C interaction persists in the radical state, suggesting substantial bond elongation rather than complete dissociation of the carboxylate-containing moiety. The calculated oxidation-induced structural rearrangement may create a more favorable geometry for intermolecular C–N bond formation. Since the carboxylate group remains within the molecular structure, such a rearrangement could facilitate intermolecular coupling and the formation of longer peptide-like chains. However, such coupling should therefore be regarded as a possible consequence of the predicted structural rearrangement rather than an established reaction pathway.

Overall, the DFT results suggest that electrochemical oxidation could induce substantial structural reorganization of the adsorbed tripeptide, potentially facilitating intermolecular coupling and subsequent chain growth.

### 3.3. Chemical Characterization and Morphology of Ti/Collagen SAM and Ti/Collagen CV Coatings

*ATR-FTIR analysis*. To verify the presence of collagen on the Ti surface, the surfaces were analyzed by ATR-FTIR spectroscopy, and the results are shown in Figure 3. In addition to the modified Ti surfaces, Ti/collagen SAM and Ti/collagen CV, a bare Ti surface was also analyzed as a reference sample. Compared with bare Ti, both collagen-modified surfaces show a complex, very similar response, with characteristic absorption bands assigned to the collagen structure. The observed bands correspond to the characteristic amide bands A_A_, A_I_, A_II_, A_III_, A_IV_, and A_V_, as previously described [19,38,39].

Briefly, the amide A_A_, A_I_, A_II_, A_III_, A_IV_, and A_V_ bands were observed at approximately 3298, 1646, 1549, 1240, 551, and 622 cm^−1^, respectively. The A_I_ band position corresponds well to that observed for the hydrolyzed collagen powder used for coating preparation [19]. The bands marked with * in the 1400–1200 cm^−1^ region can be assigned to CH_2_ wagging vibrations of glycine and proline [19]. The presence of these characteristic bands on both Ti/collagen SAM and Ti/collagen CV surfaces confirms the formation of collagen on the Ti surface by both preparation methods.

*FE-SEM analysis*. Having confirmed the presence of collagen coatings on the Ti surface, the influence of the formation method on the morphology of the resulting coatings was subsequently investigated by FE-SEM. Figure 4a–c show the morphology of the unmodified Ti surface as well as surfaces modified with collagen coatings, Ti/collagen SAM and Ti/collagen CV.

The images clearly show that the collagen coating, regardless of the formation method, alters the morphology of the Ti surface characterized by marks resulting from mechanical surface treatment (Figure 4a). Self-assembly produces a continuous layer with large island-like structures and pronounced cracks (Figure 4b). The formation of a continuous layer may be attributed to the prolonged exposure of the Ti surface to the HC solution for 24 h, which enables gradual adsorption and organization of collagen molecules on the surface. The cracks observed in the SAM coating may be associated with drying-induced contraction of the collagen layer after removal from the solution.

In contrast to the SAM coating, the electrochemical method produces a distinctly different morphology dominated by a fibrous collagen structure, with visible fiber overlapping and the formation of a network-like structure (Figure 4c). The fibers are elongated and, in some areas, appear to coalesce into thicker structures. The resulting morphology may be related to the applied potential and repetitive cycling, which may affect not only the transport of collagen molecules to the surface and their organization into a fibrous structure but also the subsequent association and reorganization of the fibers into network-like and thicker structures [40]. The formation of collagen-derived fibrillar structures on Ti is also consistent with our previous potentiostatic deposition study [19], although the present CV approach differs fundamentally in the use of repeated potential cycling during coating formation.

Recent studies have shown that fibrous collagen architecture on metallic substrates is difficult to achieve without cross-linking agents or additional chemical or processing steps [41,42]. In contrast to previously reported electrically assisted approaches, including electrophoretic deposition followed by biomimetic mineralization [17] and alternating-potential electrodeposition over a substantially wider potential range [18], the present CV approach produced a distinct fibrillar network directly from an aqueous hydrolyzed collagen solution within the comparatively narrow potential range of −0.3 to +0.1 V. Therefore, the results obtained are of particular importance, as a fibrous collagen coating was formed by a relatively simple electrochemical process from an aqueous solution of hydrolyzed collagen, without the use of additional chemicals or complex processing steps.

*EDS analysis*. Complementary evidence for the presence of collagen was obtained by EDS analysis, and the results are shown in Figure 5. The EDS spectrum of bare Ti was dominated by the Ti signal, as expected. For the collagen-modified surfaces, measurements were performed at 5 kV specifically to enhance surface sensitivity. Both Ti/collagen SAM and Ti/collagen CV showed detectable carbon, nitrogen, and oxygen signals, consistent with the presence of the collagen coating.

### 3.4. Characterization of Collagen-Modified Ti Samples After Immersion in Fusayama Artificial Saliva

To investigate the behavior of collagen-modified Ti samples in a model oral electrolyte, the samples were immersed in Fusayama artificial saliva at room temperature for 7 days. After this period, the samples were removed, air-dried, and subjected to chemical, morphological, phase, and electrochemical characterization using ATR-FTIR, EDS, FE-SEM, XRD, and EIS. To determine the influence of the collagen coating, the same characterization was performed under identical conditions on bare Ti.

*ATR-FTIR analysis*. The results of the chemical characterization performed by ATR-FTIR are shown in Figure 6.

As shown in Figure 6, after 7 days of immersion in Fusayama artificial saliva, collagen-related bands remained detectable on both modified surfaces. In particular, the characteristic A_I_ band at approximately 1630 cm^−1^ and the band associated with CH_2_ vibrations of glycine and proline, marked with *, at approximately 1460 cm^−1^ were observed (see Figure 3). At the same time, new bands appeared in the spectra of all three samples, indicating the formation of new surface species during immersion. The observed bands can be associated with calcium phosphate (CaP) phases, consistent with the formation of CaP-containing surface deposits on all investigated Ti surfaces [43]. In addition, the broad band at approximately 3353 cm^−1^ for the Ti/collagen CV and 3372 cm^−1^ for the Ti/collagen SAM can be associated with O–H stretching vibrations of hydrogen-bonded water, while the band around 1660 cm^−1^ can be attributed to the bending vibration of adsorbed water molecules [39].

The ATR-FTIR results indicate that the collagen material remained present after 7 days of immersion, regardless of the formation method, and simultaneously indicate the formation of a CaP deposit on both unmodified and collagen-modified Ti. Notably, the CaP-related bands are more pronounced for the collagen-modified surfaces than for bare Ti, suggesting that collagen may favor CaP formation under the investigated immersion conditions.

*FE-SEM analysis*. The microstructural features of the CaP-containing surface layers formed on all investigated samples after 7 days of immersion in Fusayama artificial saliva were investigated by FE-SEM, and the corresponding images are shown in Figure 7.

The FE-SEM images reveal differences in the morphology and distribution of the CaP layers formed after 7 days of immersion. On bare Ti, CaP deposits appear as localized irregular structures distributed over the mechanically treated Ti surface. In contrast, the collagen-modified surfaces show more extensive coverage by CaP deposits, which is consistent with the more pronounced bands visible in the ATR-FTIR spectra (Figure 6). For the Ti/collagen SAM, the initially continuous collagen layer (Figure 4b) undergoes pronounced morphological reorganization during immersion. The collagen-related material appears to accumulate locally into thicker structures (Figure 7d, inset; supported by EDS, Figure 8d), while areas of the underlying Ti surface remain exposed between these SAM- and CaP-structures. The CaP deposits are predominantly observed on the exposed Ti surface areas.

A distinct morphology is observed for Ti/collagen CV. The fibrillar collagen structure remains clearly recognizable after 7 days of immersion, while CaP-containing deposits appear to preferentially form along the fibrillar structure and partially cover the collagen fibers (Figure 7e,f). This association of the mineral deposits with the collagen fibrillar structure is consistent with the previously reported role of collagen functional groups in promoting the adsorption of calcium and phosphate species and facilitating subsequent mineral formation [19]. The rod-like/needle-like morphology of the CaP deposits can be observed on both collagen-modified surfaces, although the overall organization and distribution of the deposits differ between the Ti/collagen SAM and Ti/collagen CV surfaces. The inset in Figure 7f shows a cluster of needle-like particles forming the CaP-containing deposit. Based on the literature, this morphology can be related to hydroxyapatite formation, while hydroxyapatite with hexagonal symmetry preferentially grows along the c-axis and may exhibit plate-like or needle-like morphologies when precipitated without a template [44].

Overall, the SEM results indicate that the collagen coatings do not remain unchanged during immersion. Instead, both coatings undergo morphological reorganization accompanied by CaP formation. While the SAM coating undergoes local reorganization and partial detachment from the Ti surface during immersion, the electrochemically prepared coating partially retains its initial fibrillar morphology, which becomes embedded within the newly formed CaP layer.

*EDS analysis.* Detailed elemental analysis of the CaP deposits formed on bare and collagen-modified Ti surfaces was performed by EDS analysis (Figure 8).

The CaP deposits identified on all investigated surfaces contain Ca, P and O, together with Na, K and Cl in varying proportions. These results are consistent with the ATR-FTIR and FE-SEM data, supporting the presence of CaP deposits on all surfaces. In addition, nitrogen was detected on both collagen-modified surfaces, providing further evidence of detectable collagen elements after 7 days of immersion. The average Ca/P atomic ratios obtained from three measurements were 1.01 for bare Ti, 1.44 for Ti/collagen SAM and 1.39 for Ti/collagen CV. Thus, both collagen-modified surfaces exhibited higher Ca/P ratios than bare Ti, with comparable values for the two coating formation methods.

For the Ti/collagen SAM surface, two representative EDS spectra were recorded from distinct surface regions (Figure 8c,d). The spectrum obtained from the CaP-covered region (Figure 7c) shows the presence of Ca and P, whereas the spectrum recorded from the detached, collagen-rich structure shown in the SEM inset (Figure 7d) is dominated by C and O, with a detectable N signal (~5 at.%) and no detectable Ca or P. The elemental composition of this structure further supports its assignment as a collagen-containing residue remaining on the Ti surface after immersion. Thus, the EDS results indicate that a fraction of the collagen-rich material on the SAM surface remained spatially separated from the CaP deposit.

In contrast, the fibrillar structure of the Ti/collagen CV coating remained closely associated with the CaP deposits after immersion, as observed by FE-SEM (Figure 7e,f) and supported by the corresponding EDS results (Figure 8b). Thus, the electrochemically formed coating appears to provide a closer spatial association between the retained collagen fibrillar framework and the newly formed inorganic CaP phase.

*XRD analysis*. The phase composition of the CaP deposits formed on the investigated samples after 7 days of immersion in Fusayama artificial saliva was analyzed by XRD, and the results are shown in Figure 9. All diffraction patterns are dominated by the reflections of the Ti substrate (ICDD PDF 00-044-1294) [45], which is expected given the low thickness of the surface deposits. Nevertheless, additional reflections associated with the newly formed CaP phases can be identified in the enlarged 20–36° 2*θ* region.

For bare Ti, additional reflections at approximately 28.8° and 32.1° 2*θ* were observed and can be assigned to β-calcium pyrophosphate (β-Ca_2_P_2_O_7_; ICDD PDF 00-009-0346) [45]. In contrast, the collagen-modified surfaces exhibited reflections consistent with an HAp-related phase (HAp; ICDD PDF 00-009-0432) [45]. For Ti/collagen SAM, reflections were observed at approximately 26.1°, 31.9°, and 32.5° 2*θ*, while the Ti/collagen CV surface showed reflections at approximately 25.8°, 26.2°, 32.0°, 32.4°, and 33.0° 2*θ*.

Overall, the XRD results support the presence of an apatite-related phase on the collagen-modified surfaces. The sub-stoichiometric Ca/P ratios measured by EDS are consistent with a calcium-deficient apatite composition but do not independently establish the stoichiometry of the mineral phase. The relatively broad diffraction features may indicate low crystallinity and structural disorder of the apatite-related deposits [46]. In addition, because the diffraction patterns are strongly dominated by the Ti substrate and the amount of surface deposits is relatively low, the presence of minor, poorly crystalline, or X-ray-amorphous CaP phases cannot be excluded.

The formation of apatite-related CaP deposits after 7 days of immersion indicates that the collagen-modified titanium surfaces supported spontaneous mineral deposition in the model oral electrolyte. Collagen may provide an organic matrix capable of facilitating Ca^2+^ binding and creating favorable local conditions for calcium phosphate nucleation.

**Figure 10:**
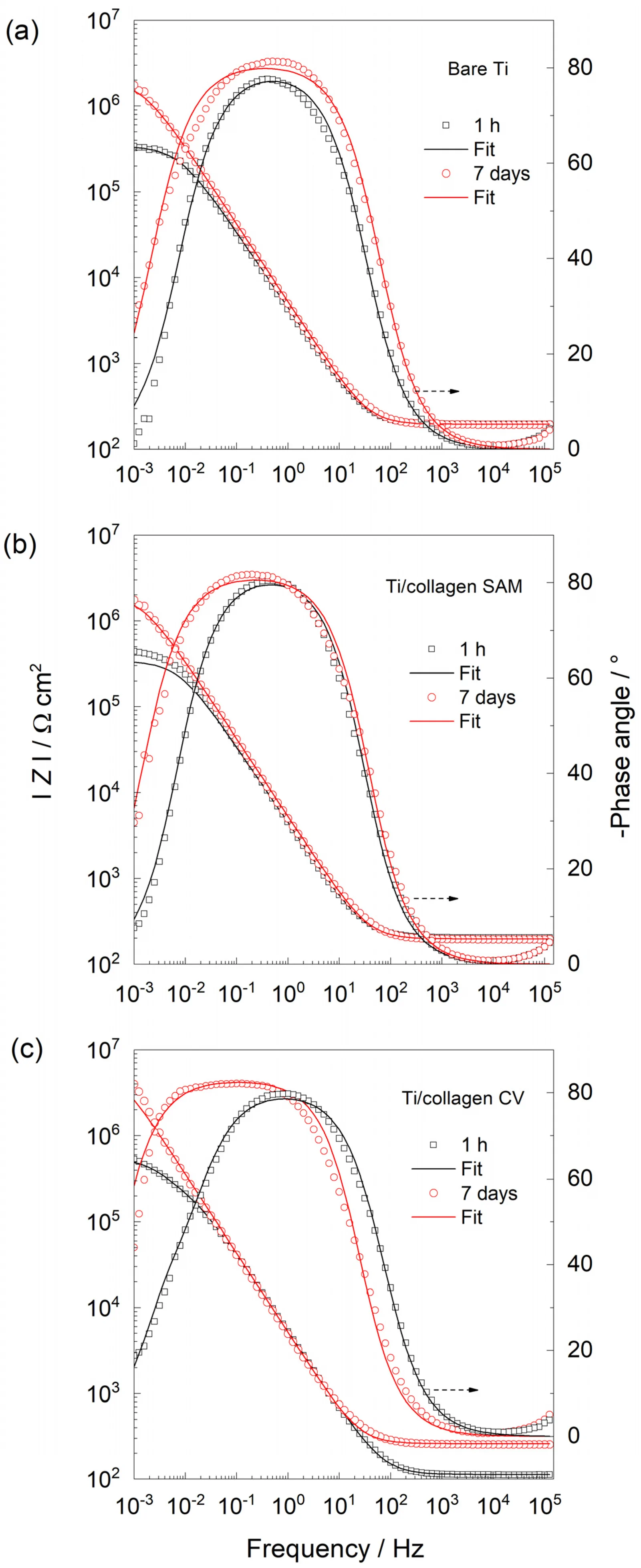
Bode plots showing the impedance modulus and phase angle of (**a**) bare Ti, (**b**) Ti/collagen SAM, and (**c**) Ti/collagen CV during immersion in Fusayama artificial saliva. Experimental data (symbols) recorded after 1 h and 7 days of immersion are shown together with the corresponding fits (lines).

**Figure 1:**
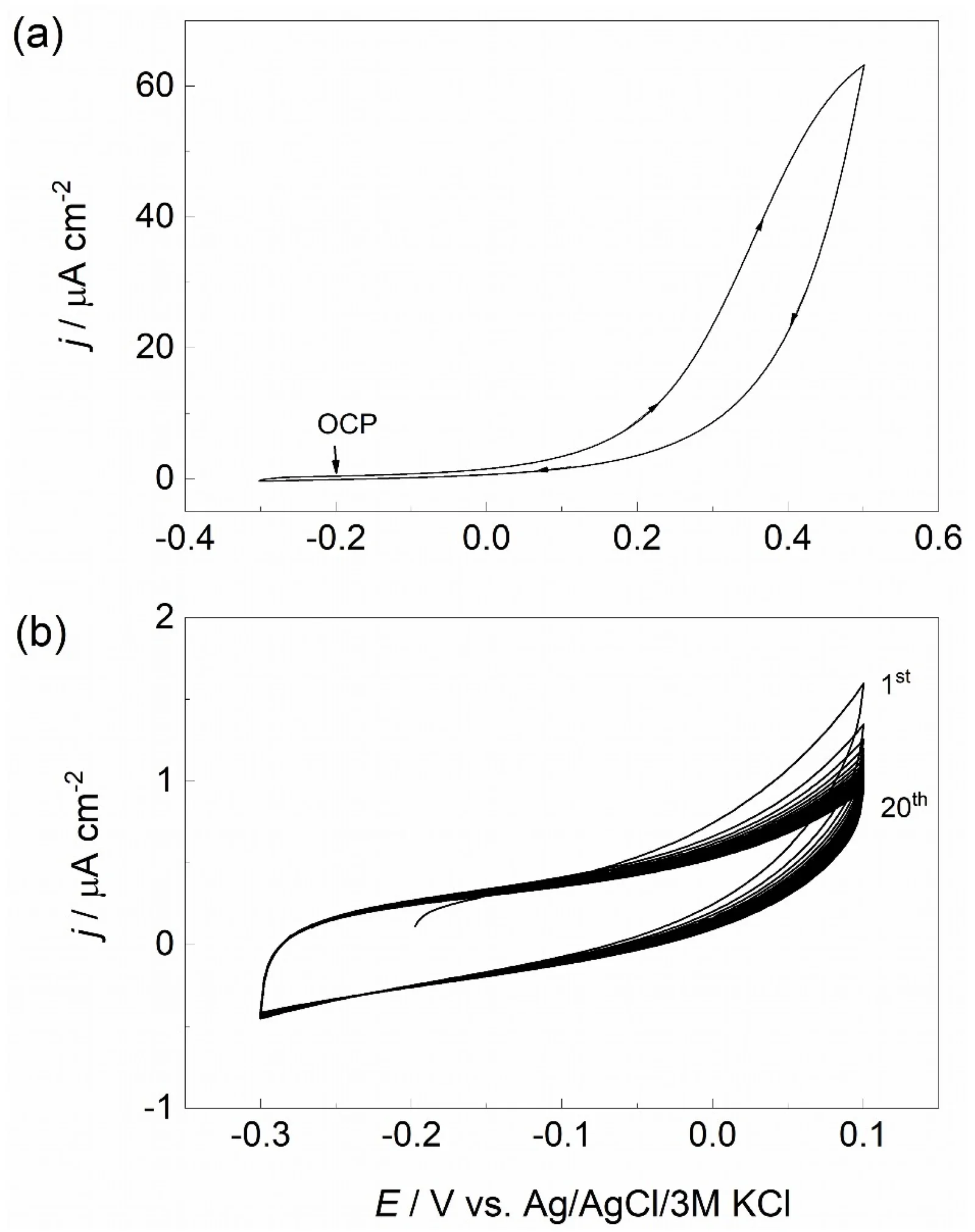
Cyclic voltammetric responses of Ti in the HC solution: (**a**) cyclic voltammogram recorded over a wider potential range at a scan rate of 50 mV s^−1^; (**b**) evolution of the cyclic voltammetric response during 20 cycles within the selected coating-formation potential range at a scan rate of 10 mV s^−1^.

**Figure 2:**
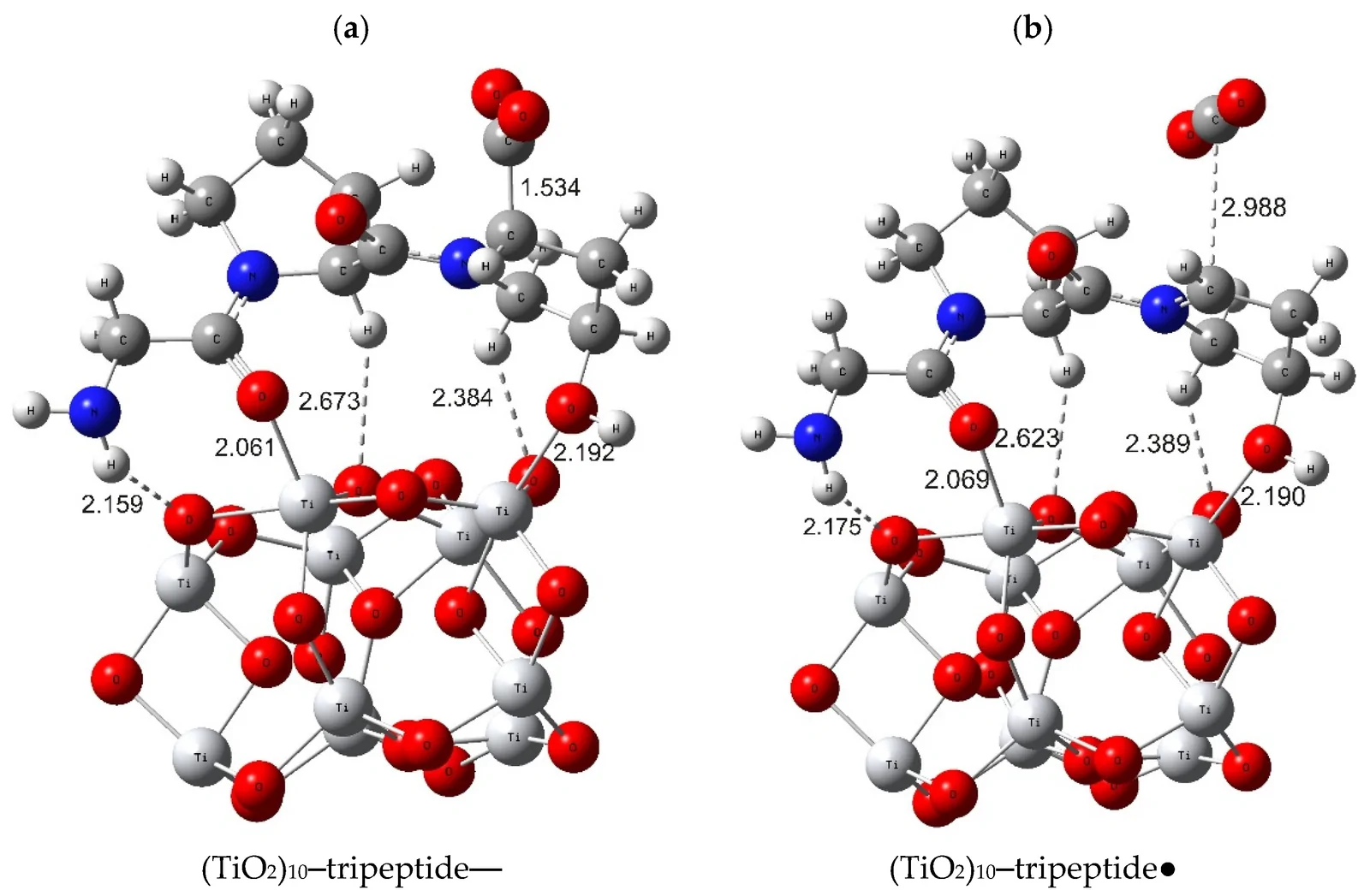
Optimized structures of the (**a**) anionic tripeptide adsorbed on the (TiO_2_)_10_ cluster, (TiO_2_)_10_–tripeptide^−^, and the (**b**) corresponding neutral radical, (TiO_2_)_10_–tripeptide^●^, formed upon one-electron oxidation. Selected bond lengths are indicated in Å. Color code: Ti—light gray; O—red; C—gray; N—blue; H—white.

**Figure 3:**
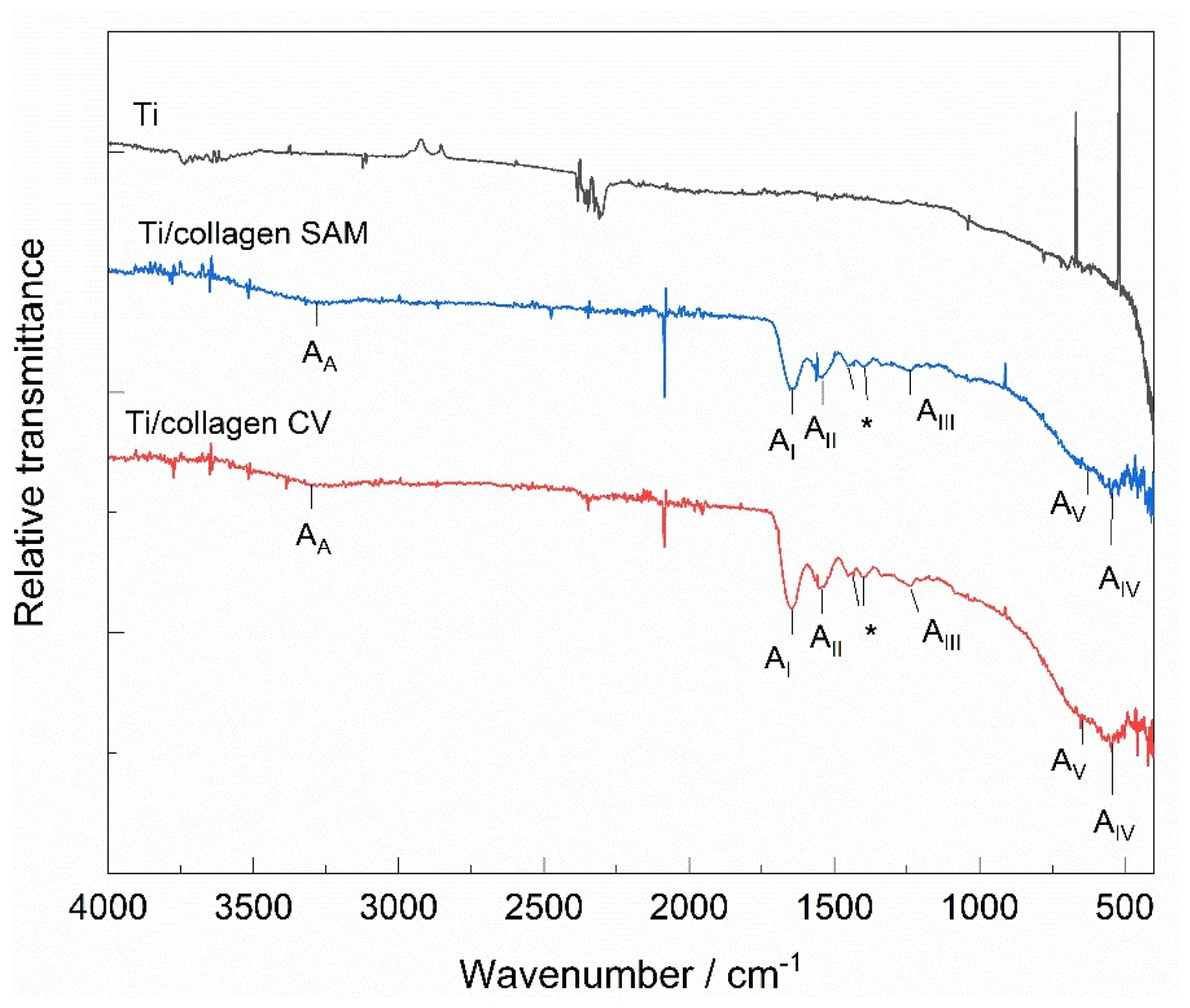
ATR-FTIR spectra of bare Ti and Ti modified with collagen prepared by self-assembly (Ti/collagen SAM) and cyclic voltammetry (Ti/collagen CV). The bands marked with * can be assigned to CH_2_ wagging vibrations of glycine and proline.

**Figure 4:**
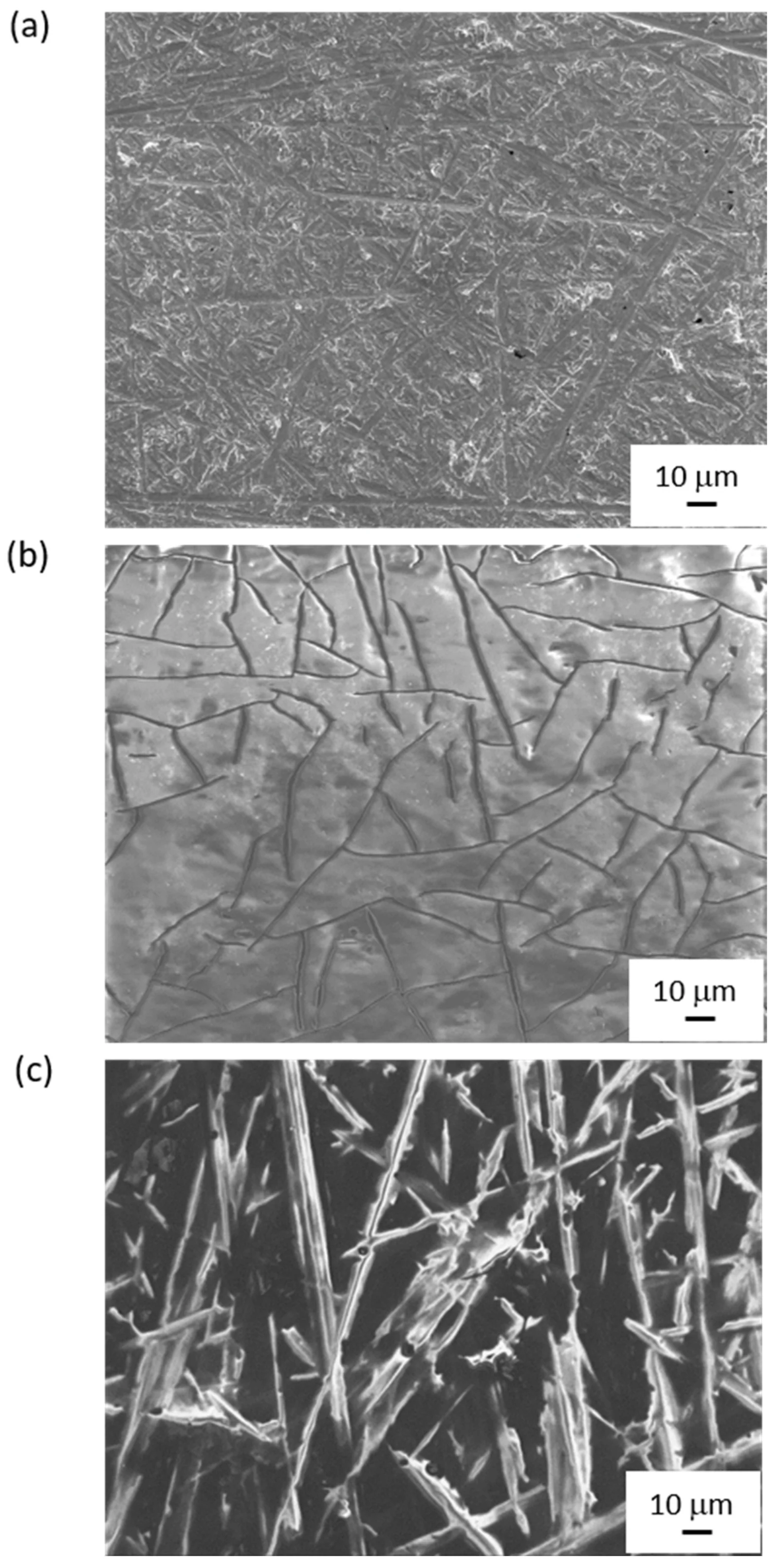
FE-SEM images of (**a**) the bare Ti surface and Ti surfaces modified with collagen prepared by (**b**) self-assembly (Ti/collagen SAM) and (**c**) cyclic voltammetry (Ti/collagen CV). The micrographs were recorded at an accelerating voltage of 5 kV and a magnification of 500×.

**Figure 5:**
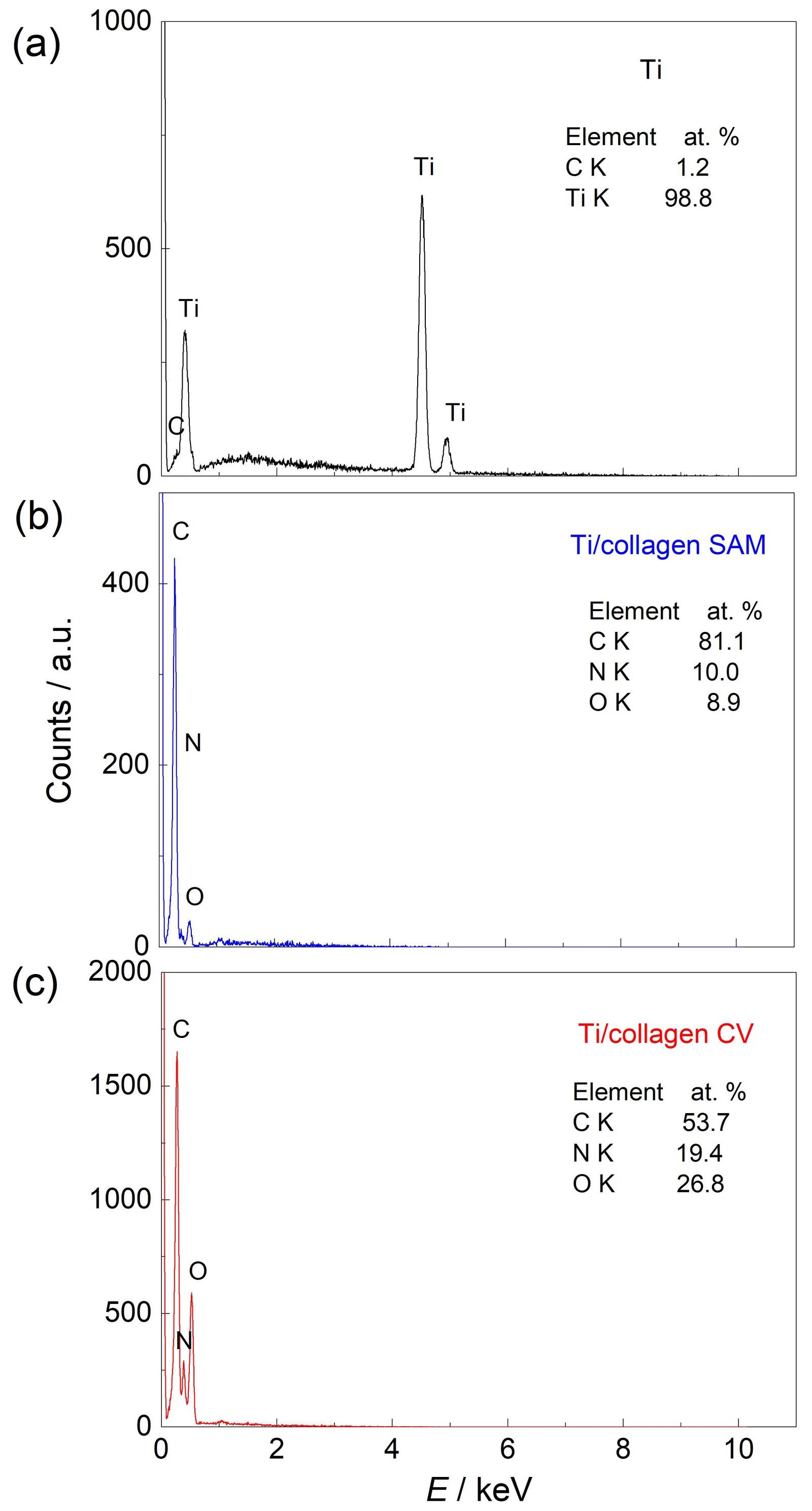
EDS spectra of (**a**) bare Ti, (**b**) (Ti/collagen SAM), and (**c**) (Ti/collagen CV) surfaces.

**Figure 6:**
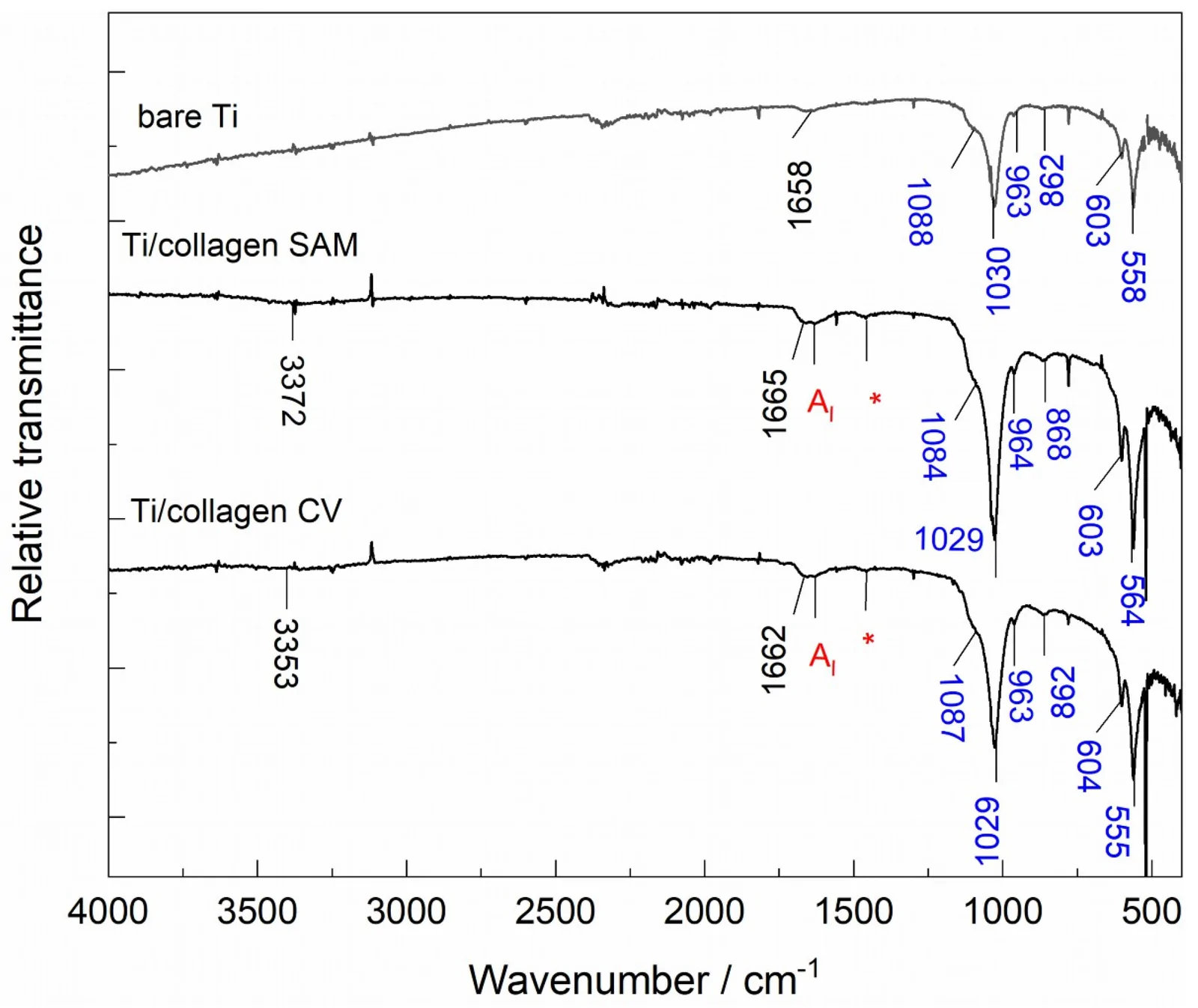
ATR-FTIR spectra of bare Ti and Ti modified with collagen prepared by self-assembly (Ti/collagen SAM) and cyclic voltammetry (Ti/collagen CV), recorded after immersion in Fusayama artificial saliva. Collagen-related bands are highlighted in red, CaP-related bands in blue, and water-related bands in black. The bands marked with * can be assigned to CH_2_ wagging vibrations of glycine and proline.

*EIS analysis*. Because the processes occurring at the Ti–electrolyte interface are electrochemical in nature, it was important to investigate the electrochemical behavior of collagen-modified Ti samples during immersion in a model oral electrolyte of artificial saliva. Specifically, the aim was to determine how the collagen coating changed the interface between Ti and saliva and whether its presence provided additional barrier properties to the titanium. The electrochemical behavior was investigated by EIS over a wide frequency range at the OCP at room temperature during continuous immersion in Fusayama artificial saliva for 7 days. Two immersion times were selected for detailed analysis: after 1 h and after 7 days of immersion. Before the first EIS measurement, the system was allowed to stabilize at OCP for 1 h. The EIS spectra, in the form of Bode plots, are presented in Figure 10. EIS measurements were performed on three independently prepared samples for each experimental group (Ti, Ti/collagen SAM, and Ti/collagen CV), and comparable impedance responses were obtained within each group.

**Table 1:** EIS fitting parameters obtained for bare Ti, Ti/collagen SAM and Ti/collagen CV after 1 h and 7 days of immersion in Fusayama artificial saliva.

Samples	*R*_s_/ Ω cm^2^	*Q*_1_ × 10^6^/ Ω^−1^ cm^−2^ s^n1^	n_1_	*R*_1_/ MΩ cm^2^	*Q*_2_ × 10^6^/ Ω^−1^ cm^−2^ s^n2^	n_2_	*R*_2_/ MΩ cm^2^	χ^2^ × 10^−3^
**bare Ti**								
**1 h**	197	44.2	0.888	0.35				2.0
**7 days**	130	36.9	0.900	1.29	/			1.4
**Ti/collagen SAM**								
**1 h**	205	40.4	0.912	0.43	/	/	/	1.5
**7 days**	196	36.1	0.908	1.99	/	/	/	2.8
**Ti/collagen CV**								
**1 h**	113	36.4	0.897	0.33	136	0.909	0.21	1.1
**7 days**	260	35.7	0.924	6.20	/	/	/	1.7

Electrical equivalent circuits (EECs) were used to describe the structure of the Ti/artificial saliva and Ti/collagen/artificial saliva interfaces, and the corresponding modeling results are given in Table 1. Due to the microscopic inhomogeneities of the systems, a constant phase element (CPE) was used instead of an ideal capacitor (C) [47]. For most conditions investigated, the spectra were adequately described by an *R*s(*Q*_1_*R*_1_) circuit, whereas the Ti/collagen CV surface after 1 h required an additional *R*_2_*C*_2_ relaxation, giving an *R*s(*Q*_1_[*R*_1_(*Q*_2_*R*_2_)]) response [48]. *R*_s_ represents the electrolyte resistance.

The EIS responses reveal changes in the interfacial behaviour depending on increasing immersion time for all samples investigated. For bare Ti, after just 1 h of immersion, the surface already exhibits relatively good barrier properties, as indicated by the relatively high ∣*Z*∣ values, which can be attributed to the presence of the spontaneously formed TiO_2_-rich passive oxide layer [49]. After 7 days of immersion, the barrier properties further improve, as indicated by the increase in ∣Z∣ and broadening of the medium-frequency phase-angle response. This behavior can be associated with further development of the passive layer and the simultaneous formation of a CaP phase on the surface, as confirmed by FE-SEM, EDS, XRD and ATR-FTIR. The resulting surface film, consisting of the passive oxide layer and newly formed CaP phase, contributes to the enhanced barrier properties of the surface. In this model, *R*_1_ represents the effective polarization resistance associated with the Ti/passive layer/Fusayama saliva interface after 1 h and with the Ti/(passive layer + CaP)/saliva interface after 7 days, while *Q*_1_ describes its non-ideal capacitive behavior.

**Figure 7:**
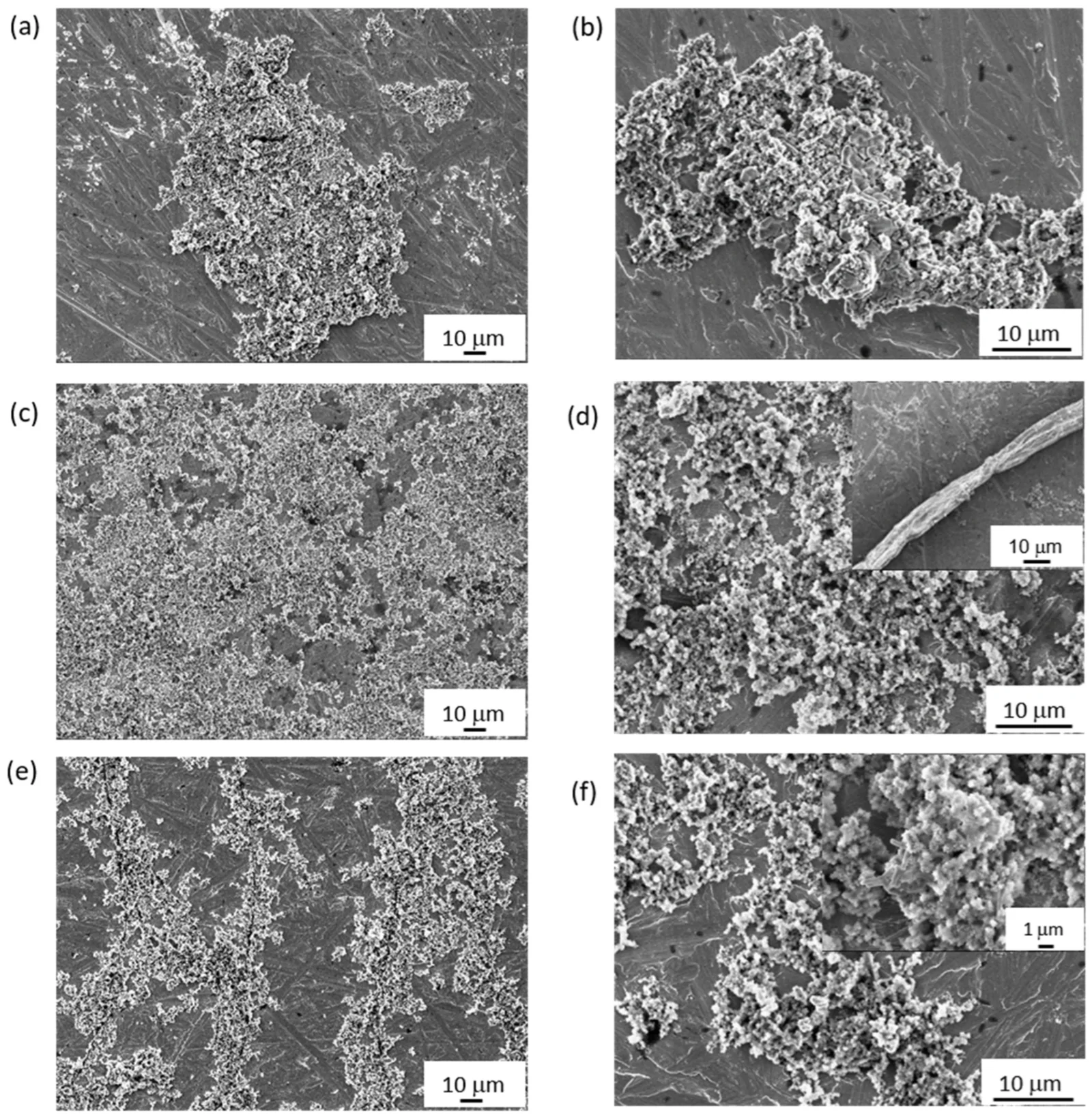
FE-SEM images recorded after 7 days of immersion in Fusayama artificial saliva of (**a**,**b**) bare Ti, (**c**,**d**) Ti/collagen SAM, and (**e**,**f**) Ti/collagen CV. The inset in (**d**) shows a detached portion of the collagen coating on the Ti/collagen SAM surface, while the inset in (**f**) shows a representative cluster of needle-like CaP deposits.

**Figure 8:**
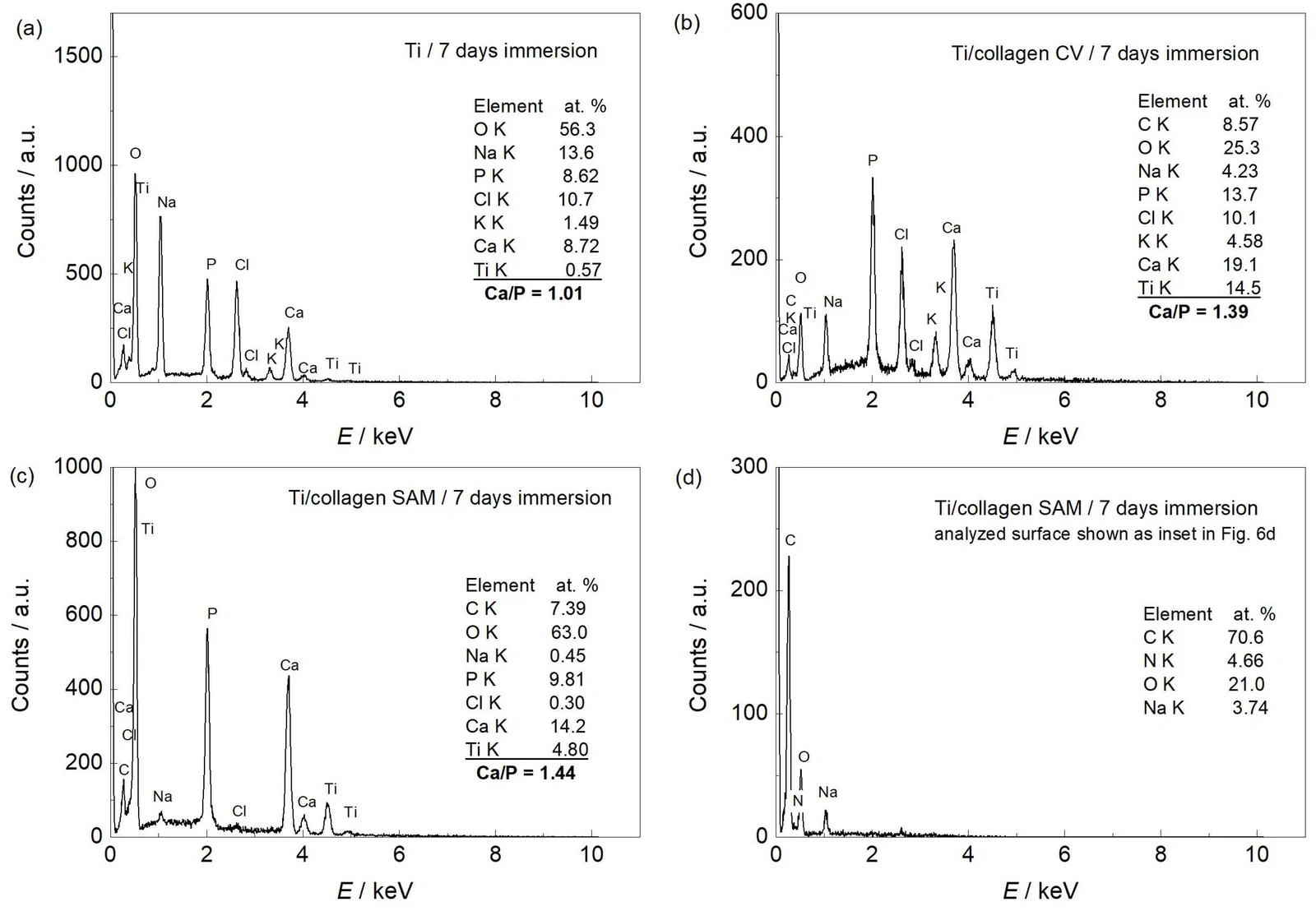
EDS spectra of the investigated samples after 7 days of immersion in Fusayama artificial saliva: (**a**) bare Ti, (**b**) Ti/collagen CV, and (**c**,**d**) Ti/collagen SAM surfaces. The spectrum shown in (**c**) represents the CaP deposits, whereas the spectrum shown in (**d**) represents a detached portion of the collagen coating shown as an inset in Figure 7d.

**Figure 9:**
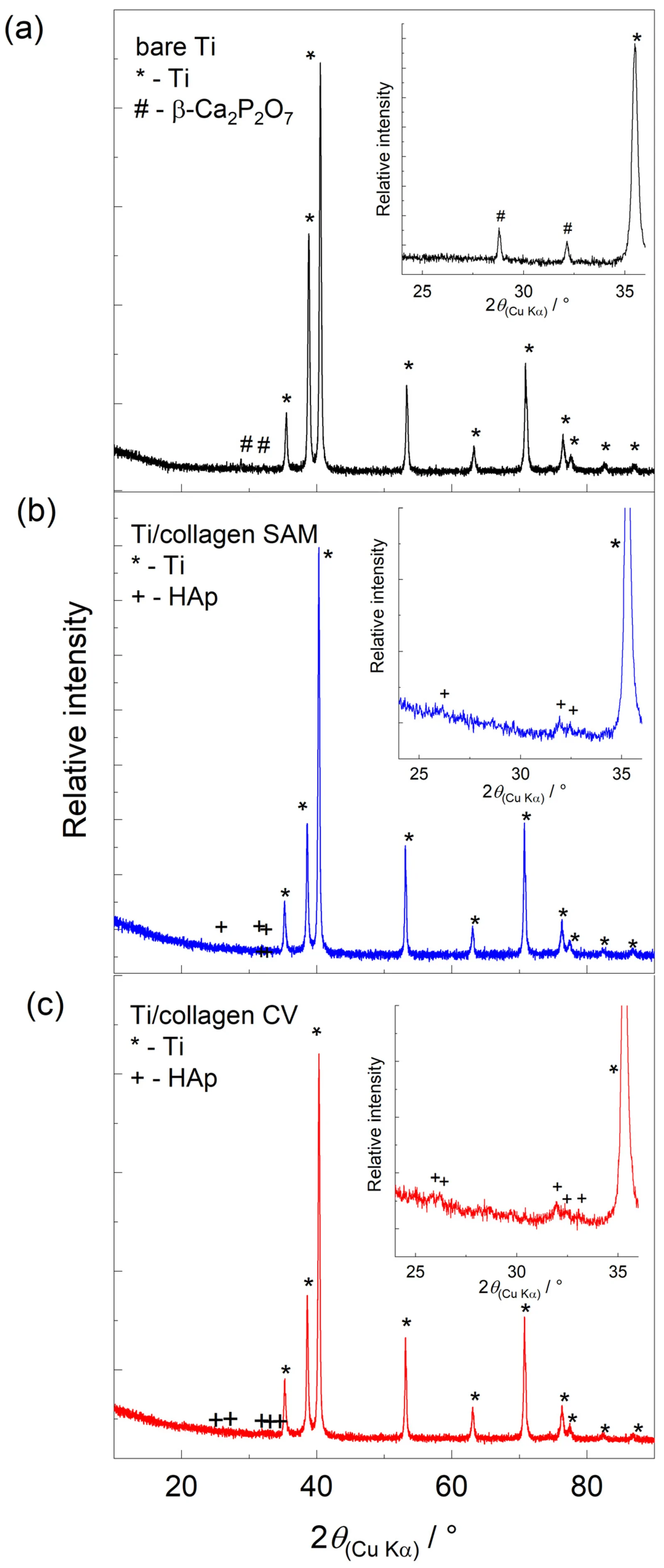
Diffractograms recorded in the 2*θ* range from 10 to 90° for (**a**) bare Ti, (**b**) Ti/collagen SAM, and (**c**) Ti/collagen CV. In each panel, the diffractogram of the corresponding sample recorded in the narrower 2*θ* range from 20 to 36° is shown as an inset. Phases are marked with symbols on the diffractograms: *—Ti (ICDD PDF 00-044-1294); +—hydroxyapatite, HAp (ICDD PDF 00-009-0432); and #—β-Ca_2_P_2_O_7_ (ICDD PDF 00-009-0346).

In the case of the Ti/collagen SAM sample, the polarization resistance also increases substantially over the 7-day immersion period, as shown in Figure 10b. During the initial immersion period (after 1 h), the collagen coating may undergo hydration and reorganization in contact with the saliva, resulting, after 7 days, in local accumulation of collagen-rich material, as seen in the SEM results (inset in Figure 7d). With prolonged immersion (after 7 days), a CaP deposit is formed on the surface, as demonstrated by XRD, ATR-FTIR, and EDS. The presence of this new phase is also reflected in the EIS response by the marked increase in ∣*Z*∣, corresponding to an increase in *R*_1_ (Table 1), and the broadening of the phase-angle response.

As for bare Ti, the EIS spectra of the Ti/collagen SAM surface were adequately described by a simple EEC. In this model, *R*_1_ represents the effective polarization resistance associated with the Ti/collagen/Fusayama saliva interface after 1 h and the Ti/(collagen + CaP)/Fusayama saliva interface after 7 days, while *Q*_1_ describes its non-ideal capacitive behavior. The marked increase in the resistance component of the Ti/collagen SAM surface during prolonged immersion from 0.43 MΩ cm^2^ (1 h) to 1.99 MΩ cm^2^ (7 days) can be associated with the formation and growth of the new CaP phase. As shown by the FE-SEM and EDS results, a substantial fraction of the collagen-rich material becomes spatially separated from the CaP deposits after immersion. Thus, the contribution of the remaining collagen to the overall barrier properties appears to be less pronounced than that of the newly formed CaP phase.

The EIS results clearly show that the change in electrochemical response with immersion time in Fusayama artificial saliva is most pronounced for the Ti/collagen CV sample (Figure 10c). As shown by the SEM results, the starting surface of this sample is covered with a porous network of collagen fibers. Therefore, the EIS data obtained after 1 h immersion were described using an EEC with two time constants. The higher-frequency time constant (*Q*_1_*R*_1_) can be associated with the electrochemical response of the outer fibrillar collagen framework and its interaction with the electrolyte, whereas the lower-frequency response (*Q*_2_*R*_2_) can be associated with electrochemical processes in the inner Ti/passive layer/collagen interfacial region. After 7 days of exposure to artificial saliva, the collagen fibers remained associated with the surface and provided a framework along which the newly formed CaP deposit developed, as shown by SEM, XRD, EDS, and FTIR results. According to the EIS response, this formation of a new phase along/over the collagen fibers was reflected in a pronounced broadening of the phase-angle response and a marked increase in the barrier properties of this sample. The EIS response is then adequately described by an EEC with one time constant, representing the effective polarization resistance at the Ti/(collagen + CaP)/saliva interface and its non-ideal capacitive behavior. The total resistance (*R*_1_ + *R*_2_) of the interfacial system increased from 0.54 MΩ cm^2^ after 1 h to 6.20 MΩ cm^2^ after 7 days of immersion, corresponding to an increase of more than one order of magnitude (Table 1). This pronounced increase in barrier resistance can be related to the combined contribution of the retained collagen fibers and the CaP deposit. Thus, unlike the SAM coating, where the increase in resistance appears to be predominantly associated with CaP formation, the electrochemically formed coating retained an organic–inorganic interfacial architecture in which both collagen and the CaP contribute to the enhanced barrier properties. The high barrier resistance observed for the Ti/collagen CV surface after prolonged immersion is consistent with our previous findings for electrochemically deposited collagen-containing structures on Ti, where retention of the collagen-derived surface layer was likewise associated with increased electrochemical barrier properties in artificial saliva [19].

Overall, the results of the comprehensive multi-technique characterization clearly demonstrate that the formation method strongly influences the architecture of the collagen coating, which in turn determines its interfacial behavior during immersion in artificial saliva. The EIS results demonstrate that the investigated surfaces follow different interfacial evolution pathways during immersion. Bare Ti exhibits a relatively stable passive response, with an increase in barrier resistance associated with the development of the CaP surface layer. The Ti/collagen SAM surface also shows an increase in resistance during prolonged immersion, which appears to be mainly associated with the development of the CaP surface layer. In contrast, the Ti/collagen CV surface exhibits the highest barrier resistance after 7 days, consistent with the retention of the collagen fibers and their close association with the newly formed CaP phase. The resulting organic–inorganic surface architecture is associated with enhanced electrochemical barrier properties compared with unmodified Ti. EIS results support that electrochemical polarization promotes the formation and organization of the collagen-containing coating within a substantially shorter time.

### 3.5. Time-Dependent Ti Release and Cell Viability

**Figure 11:**
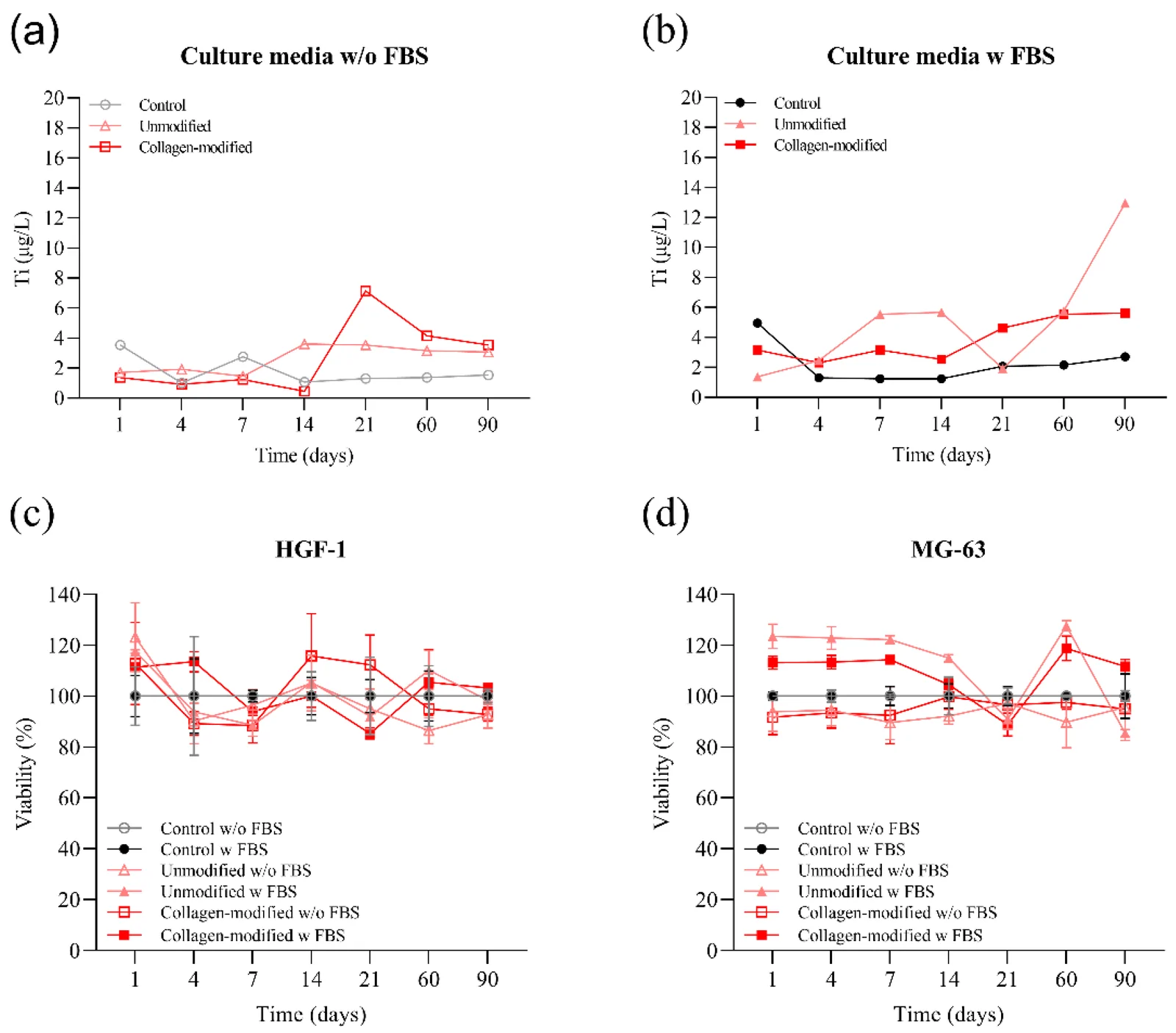
Ti release concentration over 90 days in cell culture medium (**a**) without and (**b**) supplemented with fetal bovine serum (FBS), and cell viability of (**c**) HGF-1 and (**d**) MG-63 cells after 24 h exposure to conditioned media collected during the 90-day titanium release study. Open symbols represent serum-free conditions, whereas filled symbols represent FBS-supplemented conditions. Circles indicate the control (media without Ti), triangles represent unmodified Ti, and squares represent Ti/collagen CV samples.

Titanium concentrations remained low throughout the 90-day incubation period in cell media (Figure 11a,b), with no pronounced time-dependent increase in Ti release observed for most samples. Comparably low levels of titanium release have previously been reported for titanium dental materials under simulated physiological conditions [50]. A higher Ti concentration of 12.96 µg L^−1^ was detected only in one aliquot of FBS-supplemented medium after 90 days. However, this value was substantially lower than the 20 ppm Ti concentration reported in another in vitro model to significantly reduce cell viability after 24 h exposure [51]. To assess possible biological effects, HGF-1 and MG-63 cells were exposed for 24 h to the corresponding conditioned media (Figure 11c,d). No reduction in cell viability was observed for either cell line, irrespective of incubation time, serum supplementation, or collagen modification. This is consistent with previous reports showing that adverse cellular effects of titanium are mainly observed at substantially higher Ti concentrations than those detected here [51]. Moreover, collagen modification did not affect cytocompatibility, what is in agreement with previous studies on collagen-coated titanium surfaces [52]. These results reflect indirect in vitro cytocompatibility based on cell exposure to conditioned media and do not provide information on direct cell adhesion, proliferation, or osteogenic activity on the coated surfaces.

## 4. Conclusions

Collagen coatings were successfully formed on Ti by self-assembly and electrochemical formation by cyclic voltammetry, as confirmed primarily by ATR-FTIR and supported by FE-SEM and EDS analyses.

FE-SEM showed that self-assembly yielded a relatively continuous collagen layer with locally accumulated island-like structures, whereas cyclic voltammetry produced overlapping fiber-like structures forming a distinct network-like morphology.

DFT calculations showed pronounced structural rearrangement of the adsorbed Gly–Pro–Hyp model upon one-electron oxidation, providing molecular-level insight into the redox response of collagen-derived species at the TiO_2_ interface. Because the calculated oxidation potential lies outside the potential range used for coating formation, these results are not interpreted as a direct mechanistic explanation for the experimentally observed fibrillar morphology.

Apatite-related calcium phosphate deposits formed on both collagen-modified titanium surfaces after 7 days of immersion in the model oral electrolyte. Their spatial organization differed between the two coatings, reflecting differences in the evolution of the SAM and CV surface architectures during immersion.

After 7 days of immersion, the Ti/collagen CV surface exhibited the highest barrier resistance, reaching 6.20 MΩ cm^2^, compared with 1.29 and 1.99 MΩ cm^2^ for bare Ti and Ti/collagen SAM, respectively. This behavior was associated with retention of the fiber-like coating architecture and its close spatial association with the newly formed CaP deposits.

The tested Ti and Ti/collagen CV samples exhibited low Ti release during prolonged exposure to cell culture media, while the corresponding conditioned media did not reduce the metabolic viability of human gingival fibroblasts (HGF-1) or osteoblast-like (MG-63) cells under the investigated conditions, supporting favorable indirect in vitro cytocompatibility.

## Data Availability

The original contributions presented in this study are included in the article. Further inquiries can be directed to the corresponding authors.
